# Physical and Anthropometric Characteristics Do Not Differ According to Birth Year Quartile in High-Level Junior Australian Football Players

**DOI:** 10.3390/sports9080111

**Published:** 2021-08-12

**Authors:** Paul Larkin, Carl T. Woods, Jade Haycraft, David B. Pyne

**Affiliations:** 1 Institute for Health and Sport, Victoria University, Melbourne, VIC 8001, Australia; carl.woods@vu.edu.au (C.T.W.); jade.haycraft@vu.edu.au (J.H.); 2 Maribrynong Sports Academy, Melbourne, VIC 3032, Australia; 3 Research Institute for Sport and Exercise, University of Canberra, Canberra, ACT 2617, Australia; David.Pyne@canberra.edu.au

**Keywords:** talent identification, talent development, performance assessment, relative age effect

## Abstract

The aim of this study was to explore differences in the physical fitness and anthropometric profiles between birth year quartiles of players attending the Australian Football League (AFL) National Draft Combine. Date of birth, anthropometric, 20 m sprint, vertical and running vertical jump, AFL planned agility, and 20 m Multi-Stage Fitness Test (MSFT) data were obtained for players selected to attend the Combine between 1999 and 2019 (n = 1549; M_age_ = 18.1; SD_age_ = 0.3). The underlying density distributions of the data were visually explored using violin plots overlaid with box and whisker plots. A multivariate analysis of variance (MANOVA) was then used to model the main effect of birth quartile (four levels) on the physical and anthropometric scores. Results showed that physical and anthropometric test scores did not significantly differ according to birth quartile (V = 0.008, F = 0.880, *p* = 0.631). We conclude that the physical and anthropometric profiles of high-level junior Australian Football players were similar according to birth year quartile across the modeled period. Therefore, how players utilize their physical and anthropometric attributes during game-play via contextualized, representative assessments, such as small-sided games, should be considered when examining potential causes of a RAE.

## 1. Introduction

Annual age-grouping policies are common across most team sports and involve organization of athletes into defined chronological age groups. The Australian Football League (AFL) participation pathway has two competition streams: the local participation pathway and the talent pathway that flows into the elite competition. The local participation pathway consists of age-grouped levels from Under (U) 10 years old to open age competition, with the talent pathway encompassing regional and development squads, state squads at the U14–U16 levels, and national squads at the U16–U18 levels, with players then potentially selected for a professional senior club. Players identified as talented within the local participation pathway are selected by coaches and talent managers to join their regional team to compete in state-level competitions (see Woods [1]). The relative age effect (RAE) is a demographic phenomenon involving a bias towards the selection of athletes born earlier in an age group year compared to those born later in the same year [2,3,4]. Further, the RAE is commonly observed in male invasion sports that require physical precocity, such as Australian football [3,5,6], basketball [7,8,9], ice hockey [10,11,12], rugby [13,14,15], and soccer [4,16,17,18,19,20,21,22].

Talent in sport is not dependent on birth month, though, but rather on a complex interaction of multifactorial performance attributes including physiological, technical, tactical, psychological, and sociological influences [23,24]. The RAE has been reported to occur early in the development pathways of junior athletes in invasion sports, implying a bias towards the selection of early maturing athletes over late-maturing talented athletes [5,25,26,27,28]. Physiological growth and maturation have been proposed as the underlying mechanism for the RAE in invasion sport development pathways [25], with the superior physicality of early maturing players often confused as talent by coaches and talent scouts [6]. However, the chronological age of players may have a greater impact on an athlete’s playing experience, perceptual and motor skills, and social and psychological development, more than their physiological maturation [29].

Additionally, in popular team invasion sports, such as Australian football, which have a large grass-root participation base, the RAE may be amplified across key stages of development, given the high participation rates, creating selection pressure at both local participation and talent levels [30]. In Australian football, specifically, the RAE has been reported within the U10-U12 competitions [6], amplifying as players are selected into the talent pathway [5,6,28]. To mitigate the RAE within the AFL talent pathway, two rule changes to the age-policy of National U18 draft attendees in 2003 and 2009 were implemented. While policy changes removed birth month bias in the first half of the selection year, the RAE was still evident in the first and last quarter of the selection calendar [28]. Selection bias also occurred at the State U16 level and likely carried through to the National Draft level (i.e., U18), making age-policy changes at the National U18 level irrelevant [28]. In mature-aged AFL draftees (>20 years-old), birth month bias was reversed, with 63% of mature-aged players drafted to elite AFL teams having been born in the latter half of the year [3]. Therefore, given this known RAE in Australian football [28] it is possible that the physical fitness and anthropometric attributes of junior (U18) talent selected Australian football players is influenced by their birth quartile, manifesting in the noted selection bias. This distribution is, however, yet to be examined.

Despite increased interest and proposed solutions to address the issue, researchers acknowledge there is limited impact of research outcomes on talent selection practices [31]. Current talent selection practices within the AFL talent pathway involve coaches and selectors inviting talent identified players to attend State and National Draft Combines. AFL clubs then select players at the annual National Draft to join their club playing lists [32]. The annual National Draft Combine places a heavy focus on selected anthropometric measures (i.e., height and body mass) and physical tests including 20 m sprint, vertical and running vertical jump, AFL planned agility, and aerobic endurance, previously the 20 m Multi-Stage Fitness Test (MSFT), and now the YOYO Intermittent Recovery (IR) 2 [33,34]. Birth quartile and birth half of the selection year did not seem to have a major impact on physical and anthropometric profiles of players drafted to the AFL following their attendance at the National Draft Combine between 2010 and 2013 [5]. However, birth quartile may have a greater impact on physical and anthropometric profiles of players selected to attend the National Combine. While anthropometric and physical fitness attributes have a role in talent selection, training, and management, there is limited understanding of the implications of the physical profile of Australian football players born in different birth quartiles on the RAE over an extended period within the AFL talent pathway.

As Haycraft and colleagues [28] identified a RAE within a high-level junior sample (U18 age group) attending the National Combine, the aim of this study was to conduct a 20-year retrospective cross-sectional analysis of the AFL National Draft Combine. As the National Draft Combine places an emphasis on physical performance, this analysis would determine whether the RAE identified by Haycraft and colleagues [28] is influenced by physical and anthropometric differences between the birth year quartiles. Given the reported RAE within this U18 age group sample [28], we expected that physical and anthropometric profiles would differ relative to birth year quartile.

## 2. Materials and Methods

This study used a 20-year retrospective cross-sectional design to examine differences in the physical and anthropometric profiles between male Australian footballers born in differing age-group quartiles. Date of birth (DOB), physical fitness, and anthropometric data were obtained for U18 players who were selected to attend the AFL National Draft Combine (n = 1549; M_age_ = 18.1; SD_age_ = 0.3; M_attendees/year_ = 79; SD_attendees/year_ = 16) between 1999 and 2019. National player data were available for all years between 1999 and 2019. Players were classified into the following birth quartiles: Q1: January–March, N = 1396, 34%; Q2: April–June, N = 1068, 26%; Q3: July–September, N = 913, 23%; Q4: October–December N = 694, 17%. In 2003 and 2008, changes to age eligibility policies were imposed at the National U18 level to target the RAE of players invited to attend the AFL National Draft Combine. Specifically, between 1999 and 2003 players were required to turn 17 years of age by June 30, with this cut-off date shifting to April 30 in 2008. Player birth quartiles of those attending National U18 drafts in these years were classified based on the first month of the new age eligibility date (e.g., 2004–2008 Q1: May–July). All players 17 years of age were excluded from analysis between 2010 and 2013, as during these years the acquisition of these players was limited to trades, given the introduction of two new AFL teams [28]. The study was approved by the university’s human research ethics committee.

The following test data were provided by the AFL: anthropometric measures (standing height (cm), Typical Error (TE) = 1.0 cm; body mass (kg), TE = 1.0 kg); 20-m sprint (s), TE = 0.03 s; stationary vertical and running vertical jump (cm), TE = 1.4 cm; AFL planned agility (s), TE = 0.04 s; and aerobic endurance (20 m MSFT, measured via total distance reached, TE = 3%); with the best of three efforts recorded for all tests except the 20 m MSFT [34,35]. The 20 m sprint was performed from a standing stationary start, with time recorded using electronic timing gates. The vertical jump test was also performed from a stationary start, with the running vertical jump test requiring players to take a 5 m run up and perform three jumps off both their left and right legs. The AFL agility course was measured via a timed trial through a course of cones positioned 5 m apart and included three left and two right 90° angle turns. Aerobic endurance was measured using the 20 m MSFT, which is a repeated 20 m interval test that progressively increases speed until players are no longer capable of keeping pace [36,37]. All testing was conducted over a 3–4-day period at the conclusion of the junior competition season (October) and preceded the AFL National Draft by approximately 5–6 weeks.

### Statistical Analysis

First, the underlying distribution of each assessment relative to each year was visually inspected using overlaid density distribution plots in the ggplot2 package (Wickham, 2016) in the R computing language (R Core Team, 2019). This step enabled us to confidently pool the dataset. Next, to explore the underlying density distributions of the data, violin plots with an overlaid box and whisker plot (showing the median and interquartile range, with minimum and maximum test values as jittered points) were developed for all physical and anthropometric tests relative to birth quartile. This analysis was completed using the ggplot2 package (Wickham, 2016), with the plots arranged and annotated using the cowplot package (Wilke, 2017) in the R computing language (R Core Team, 2019, Vienna, Austria). A multivariate analysis of variance (MANOVA) was then used to model the main effect of birth quartile (four levels) on the physical and anthropometric test scores. This modeling was completed using the “manova()” function, with significance set α < 0.05. Pending the outcomes of the MANOVA (noted via a significant Pillai’s Trace), univariate analysis of variance was planned to identify which (if any) of the physical and/or anthropometric tests differed significantly according to birth quartile.

## 3. Results

**Figure 1:**
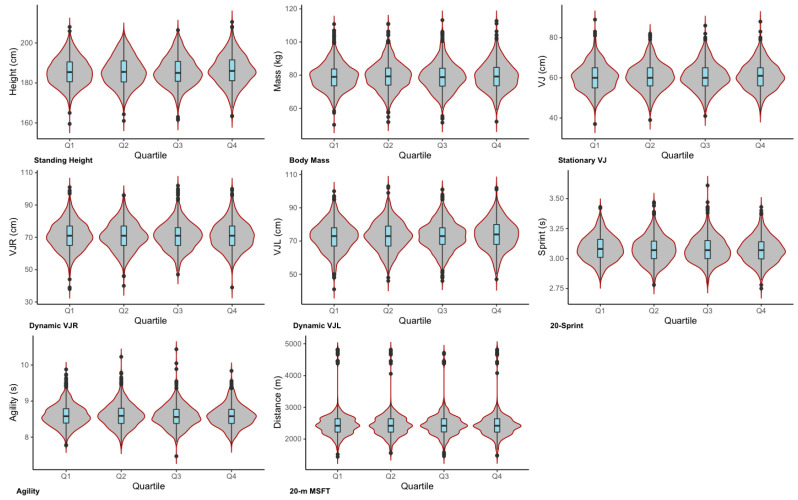
Violin plots with overlaid box and whisker plots showing the underlying density distributions of the data. Note: the ‘dots’ reflect the maximum and minimum test scores, “VJR” denotes vertical jump right leg take-off, and “VJL” denotes vertical jump left leg take-off.

Visual inspection of the density distributions of the data using the violin plots (Figure 1) indicates that physical and anthropometric test scores do not appear to meaningfully differ relative to birth quartile. In support of this observation, the MANOVA did not yield a significant Pillai’s Trace (V = 0.008, F = 0.880, *p* = 0.631), indicating the physical and anthropometric profiles of players were similar across all four birth quartiles. Given this outcome, follow up univariate analysis was not performed on the data. Mean data for the physical and anthropometric characteristics relative to birth quartile are provided in the online Supplementary Materials.

## 4. Discussion

The aim of this study was to explore if the physical and anthropometric attributes of players attending the AFL National Draft Combine differed according to birth quartile. Results did not yield a significant effect of birth quartile, with all players at this U18 level possessing a relatively homogenous physical and anthropometric profile. Therefore, it appears that players are largely selected for the AFL National Draft Combine based on relatively homogenized physical and anthropometric profiles, supporting previous studies which have suggested this occurs in professional adult populations [38,39]. Therefore, the RAE in this population may manifest in technical and tactical skills, not necessarily physical precocity.

Several studies have investigated the prevalence of RAE within talent development programs and elite senior competitions of invasion sports [3,4,5,19,40]. However, there has been limited investigation into the physical profile differences relative to birth quartile in Australian football. Our results indicate that, over the 20-year period, there was a relatively uniform physical and anthropometric profile for high-level junior Australian football players attending the National Draft Combine. This outcome supports the earlier work of Woods, Robertson [5] who, on a smaller sample, demonstrated that physical and anthropometric assessments did not differ according to birth year quartile or half year in drafted National U18 Australian football players. The RAE reported by Haycraft et al. [28] may, therefore, be the result of other factors that influence player performances, such as the technical and tactical skills needed to compete within Australian football [29], rather than physical capabilities. Taken together these data imply that, in late adolescence, early maturing athletes may not be able to maintain their physical advantages–an observation seen in soccer [37] and rugby league [13,14]. This outcome is in contradiction to several studies showing that athletes born early in a selection year are more biologically mature than those born later in the same year, with substantial differences in body size, height, strength, and power, and who may, therefore, possess an advantage during physical testing [5,28,33]. Physical and anthropometric profiles may only assist players in gaining initial access into the talent development pathway [26,28,41], with the RAE manifested by other domain-specific attributes at later stages in development. Future work in Australian football should ascertain more contextual information on how players utilize their physical and anthropometric attributes during game-play (e.g., winning the ball in a tight position and then breaking clear from the pack) to explain the observed RAE.

While the AFL National Draft Combine provides a detailed assessment of the physical and anthropometric qualities of talented U18 players [34,42], they are limited in their contextualization. This shortcoming has been highlighted by Bonney, Berry [43] who proposed that isolated, decontextualized laboratory assessments provide information on movement patterns or physical capabilities, but do not identify the proficiency of a player’s skill under match conditions within Australian football. To better understand the impact of RAE on talent development and selection in Australian football, contextualized and more representative tests should be considered, such as small-sided game assessments [44,45]. The likely benefit of incorporating such assessments is the integration of both physical and technical components in game-like contexts [36,43]. Future work should investigate whether incorporating a small-sided game assessment into the Australian football and other sporting talent pathways mitigates the RAE on talent development and selection [28].

While this study examined the influence of physical and anthropometric profiles on RAE at the National Draft Combine over a substantial time period, the outcomes should be considered with respect to limitations. A key limitation of the current study is the representativeness of the cohort analyzed. We acknowledge that the athletes assessed at the National Combine are selected to attend this event by coaches and selectors. Therefore, there is potential for an inherent bias within the sample based on how the players were invited to participate in the testing Combine. Further, while not a specific aim of this study, future research should examine whether the current findings are replicated between the local competition levels (i.e., U12′s to U18′s), and the entry levels of the talent development pathway (i.e., State U15’s and U16′s). This work will establish whether players who do not exhibit a physical profile within the associated bandwidths are discriminated against in relation to selection for more high-level talent development programs.

## 5. Conclusions

This study examined the physical and anthropometric profiles of high-level junior Australian football players attending the AFL National Draft Combine according to birth year quartile over a 20-year period. There were no significant differences in the physical and anthropometric profiles across the birth year quartiles, despite there being a known RAE across the same cohort. Therefore, it appears that players are selected for the AFL National Draft Combine based on relatively homogenized physical and anthropometric profiles, and that the RAE is influenced through other aspects of performance, such as technical and tactical skill. We recommend that future work examines the performance of players attending the National Combine via contextualized, representative assessments, such as small-sided games, when examining potential causes of a RAE.

## Data Availability

Due to institutional ethics requirements the data could not be shared publicly.

## Supplementary Materials

The following are available online at https://www.mdpi.com/article/10.3390/sports9080111/s1, Table S1: Physical and anthropometric characteristics of players relative to birth quartile.
