# Supplementary material for: Physical and Anthropometric Characteristics Do Not Differ According to Birth Year Quartile in High-Level Junior Australian Football Players
Source: Sports (Basel). 2021 Aug 12;9(8):111. doi: 10.3390/sports9080111 (PMC8402445; doi:10.3390/sports9080111)
Supplement: Supplementary file 1 [file sports-09-00111-s001.zip › sports-1289253-supplementary.pdf]

sports

Supplementary Materials

Physical and Anthropometric Characteristics Do not Differ According to Birth Year Quartile in High-Level Junior Australian Football Players

Paul Larkin [1,2,*], Carl T. Woods [1], Jade Haycraft [1] and David B. Pyne [3]

[1] Institute for Health and Sport, Victoria University, Melbourne VIC 8001, Australia; carl.woods@vu.edu.au (C.T.W.); jade.haycraft@vu.edu.au (J.H.)
[2] Maribrynong Sports Academy, Melbourne VIC 3032, Australia
[3] Research Institute for Sport and Exercise, University of Canberra, Canberra ACT 2617, Australia; David.Pyne@canberra.edu.au
* Correspondence: paul.larkin@vu.edu.au

Citation: Larkin, P.; Woods, C.T.; Haycraft, J.; Pyne, D.B. Physical and Anthropometric Characteristics Do not Differ According to Birth Year Quartile in High-Level Junior Australian Football Players. *Sports* **2021**, *9*, 111. https://doi.org/10.3390/sports9080111

Publisher's Note: MDPI stays neutral with regard to jurisdictional claims in published maps and institutional affiliations.

Copyright: © 2021 by the authors. Licensee MDPI, Basel, Switzerland. This article is an open access article distributed under the terms and conditions of the Creative Commons Attribution (CC BY) license (http://creativecommons.org/licenses/by/4.0/).

Table S1. Physical and anthropometric characteristics of players relative to birth quartile.

Birth Quartile (Sample Size)	Standing Height (cm)	Body Mass (kg)	Vertical Jump (cm)	Dynamic VJR (cm)	Dynamic VJL (cm)	20 m Sprint (s)	AFL Agility (s)	20 m MSFT (m)
Q1 (1396)	185.6 ± 6.9	79.1 ± 7.8	60.3 ± 6.9	70.8 ± 8.6	72.8 ± 8.5	3.08 ± 0.10	8.60 ± 0.30	2463 ± 407
Q2 (1068)	185.8 ± 7.3	79.4 ± 8.3	60.6 ± 6.6	70.7 ± 8.5	73.1 ± 8.7	3.07 ± 0.10	8.60 ± 0.32	2439 ± 380
Q3 (913)	185.7 ± 7.2	79.1 ± 8.5	60.6 ± 6.7	70.9 ± 8.3	72.9 ± 8.7	3.08 ± 0.10	8.57 ± 0.31	2456 ± 381
Q4 (694)	186.2 ± 7.5	79.5 ± 8.4	61.0 ± 6.7	71.1 ± 8.6	73.7 ± 8.7	3.07 ± 0.10	8.58 ± 0.31	2465 ± 460

Note: VJR denotes vertical jump right leg take-off; VJL denotes vertical jump left leg take-off; AFL denotes the Australian Football League; MSFT denotes the Multi-Stage Fitness Test.
